# Highly Sensitive SOI-TFET Gas Sensor Utilizing Tailored Conducting Polymers for Selective Molecular Detection and Microbial Biosensing Integration

**DOI:** 10.3390/bios15080525

**Published:** 2025-08-11

**Authors:** Mohammad K. Anvarifard, Zeinab Ramezani

**Affiliations:** 1 Department of Engineering Sciences, Faculty of Technology and Engineering, East of Guilan, University of Guilan, Rudsar 4489163157, Iran; 2 Department of Electrical and Computer Engineering, College of Engineering, University of Miami, Miami, FL 33146, USA

**Keywords:** SOI-TFET, conducting polymers, band-to-band tunneling, sensitivity

## Abstract

We present a highly sensitive and selective gas sensor based on an advanced silicon-on-insulator tunnel field-effect transistor (SOI-TFET) architecture, enhanced through the integration of customized conducting polymers. In this design, traditional metal gates are replaced with distinct functional polymers—PPP-TOS/AcCN, PP-TOS/AcCN, PP-FE(CN)_6_^3−^/H_2_O, PPP-TCNQ-TOS/AcCN, and PPP-ClO_4_/AcCN—which enable precise molecular recognition and discrimination of various target gases. To further enhance sensitivity, the device employs an oppositely doped source region, significantly improving gate control and promoting stronger band-to-band tunneling. This structural modification amplifies sensing signals and improves noise immunity, allowing reliable detection at trace concentrations. Additionally, optimization of the subthreshold swing contributes to faster switching and response times. Thermal stability is addressed by embedding a P-type buffer layer within the buried oxide, which increases thermal conductivity and reduces lattice temperature, further stabilizing device performance. Experimental results demonstrate that the proposed sensor outperforms conventional SOI-TFET designs, exhibiting superior sensitivity and selectivity toward analytes such as methanol, chloroform, isopropanol, and hexane. Beyond gas sensing, the unique polymer-functionalized gate design enables integration of microbial biosensing capabilities, making the platform highly versatile for biochemical detection. This work offers a promising pathway toward ultra-sensitive, low-power sensing technologies for environmental monitoring, industrial safety, and medical diagnostics.

## 1. Introduction

Gas sensors are crucial in environments requiring precise detection of hazardous and traced gases, impacting fields such as industrial safety, environmental monitoring, and public health [1,2,3]. Conducting polymers have emerged as promising sensing materials due to their room temperature operation, cost-effective fabrication, and tunable chemical and physical properties [4,5]. These polymers, such as polyaniline and polypyrrole, offer fast and reversible electrical responses to gas adsorption, enabling sensitive and selective detection [6,7]. Their solution-processability nature allows integration with various device architectures, broadening their applicability to flexible and miniaturized sensor platforms [8].

FET based gas sensors have emerged as a transformative technology for real-time, miniaturized, and highly sensitive detection of diverse gases. Their research significance is underscored by the expanding need for environmental monitoring, medical diagnostics, industrial safety, and the development of artificial olfactory systems.

Recent progress is driven by the design and integration of advanced semiconducting materials, particularly two-dimensional (2D) materials, organic semiconductors, and nanostructures [9,10,11]. The integration of FETs and gas sensors within a single 2D material not only enables on-chip trace gas detection but also amplifies sensor output directly on the device, leading to compact and efficient sensor platforms. For instance, Janus monolayers and other novel 2D materials have been engineered to achieve substantial enhancements in sensitivity and selectivity for hazardous and trace gases, even under challenging environmental conditions [12].

Nanowire-based FETs represent another milestone, where one-dimensional nanostructures act as sensitive channels, and their charge transport properties are modulated by gas adsorption. These FET sensors can harness additional controls—such as gate voltage, metal decoration, localized heating, or light irradiation—to optimize their sensing characteristics, making them highly tunable and miniaturizable for integration into smart sensing networks [13].

Recent advances focus on optimizing three core sensor metrics including the following:

- Sensitivity: achieved by maximizing gas–surface interactions via high-surface-area materials and porous architectures [12,14].
- Selectivity: improved through material functionalization and circuit-level innovations [15].
- Response time and stability: local microheaters and device engineering (e.g., silicon-on-insulator CS-FET sensors) have enabled faster recovery and reliable operation at room temperature [15].

The room-temperature operation of many modern FET gas sensors offers significant energy savings and expands their deployment in portable and wearable applications. Organic FET (OFET)-based sensors, in particular, show strong promise for flexible and wearable electronics, enabling applications such as electronic skins for health monitoring and environmental assessment [14].

FET gas sensors are intrinsically suited for system-on-chip (SoC) integration due to their compatibility with complementary metal–oxide–semiconductor (CMOS) processes [12,15]. This compatibility accelerates their adoption in distributed, wireless, and IoT-oriented sensor networks. With the integration of machine learning algorithms, FET sensor arrays can discriminate between complex gas mixtures, forming the technological backbone for artificial olfactometry (electronic noses) [16].

Key research challenges remain, including long-term stability, cross-sensitivity to environmental fluctuations, and scalability of manufacturing. Nevertheless, ongoing developments in material science, device miniaturization, and integration with advanced data analytics underscore the central role of FET gas sensors as pivotal components for next-generation sensing platforms.

In parallel, silicon-on-insulator tunnel field-effect transistors (SOI-TFETs) represent an advanced class of gas sensors with intrinsic advantages such as ultra-low power consumption, steep subthreshold swing (SS), and enhanced electrostatic control [17,18,19]. SOI substrates offer excellent electrical isolation, reducing parasitic effects and enabling device scaling, while TFETs leverage band-to-band tunneling for energy-efficient switching and heightened sensitivity near threshold voltages [6,20].

However, achieving practical implementation of SOI-TFET gas sensors presents challenges. The complexity of double-gate architectures, essential for controlling short-channel effects and improving sensitivity, demands precise fabrication and meticulous interface control [21,22]. Dash et al. showed that hetero-gate dielectric SiGe/Si TFETs can improve hydrogen sensing sensitivity but involve intricate dual-gate design and process integration [22]. Thermal instability further complicates sensor reliability, degrading threshold voltage stability and noise performance under temperature fluctuations [23].

Design considerations extend to balancing sensor parameters including channel geometry, sensing layer coupling, and noise suppression [19]. Modeling and TCAD simulations provide critical insights for optimizing these factors, guiding experimental development for enhanced gas responses [19,24]. Additionally, although SOI-TFETs achieve sub-60 mV/decade subthreshold swings, their inherently low drain currents limit signal magnitude and dynamic range [25]. Band alignment engineering and heterojunction channel designs, as demonstrated by Dash et al., allows improvement of both sensitivity and current [22].

Selectivity remains a persistent challenge for SOI-TFET gas sensors, as interference from multiple gases reduces reliability in complex environments. Editorial reviews highlight the necessity of functionalizing sensing layers and exploiting combinatorial sensor arrays to enhance discrimination [25,26,27]. Conducting polymers with tailored chemical affinity, combined with doping strategies and nanoscale engineering, offer promising routes to address these issues synergistically.

Moreover, conducting polymers, such as polypyrrole and polyaniline, have proven vital not only for gas sensing but also in biosensing applications, including microbial biosensing [28,29]. Their inherent electrical conductivity, electrochemical activity, and compatibility with biological systems allow them to serve as both transducers and immobilization platforms for enzymes and cells. This dual capability enables the realization of hybrid sensors capable of detecting both chemical vapors and microbial byproducts. It forms the foundation of modern gas sensors due to their outstanding electrical conductivity, facile synthesis, and high sensitivity to volatile analytes under ambient conditions, attributes that seamlessly intersect with the requirements of biosensors and microbial sensors [29]. In electrochemical biosensors, these materials not only function as responsive sensing elements but also act as effective transducers that facilitate efficient electron transfer between biological recognition layers and underlying electrodes, resulting in enhanced sensitivity and rapid, direct readout of bio-analyte presence [30]. For microbial sensors, the same conductive matrix can immobilize microbial enzymes or entire cells, allowing the detection of specific metabolic products with high selectivity while maintaining sensor stability. The inherent versatility of conducting polymers thus enables the development of hybrid and multifunctional sensors, where chemical and biological detection pathways converge, positioning these platforms at the forefront of real-time environmental, clinical, and food safety applications [31].

The ease of producing and modifying conducting polymers enhances their usefulness in sensing devices [32]. In electrochemical biosensors, these polymers help transfer electrons efficiently between the biological sensing elements and the electrodes, improving sensitivity and allowing quick, direct measurement of the target substance. This is especially helpful for detecting microbial metabolites in real time.

For microbial sensors, conducting polymers can hold microbial enzymes or even living cells in a stable and active state, enabling the sensor to detect specific biological products with high selectivity [33]. For example, sensors can detect gases generated by bacteria during metabolism or enzymes released during microbial activity. This capability supports applications such as detecting food spoilage, monitoring water quality, or tracking pathogens.

Conducting polymers also perform well in watery and biological environments where microbes live, thanks to their stability and ability to interact with microbial membranes. This makes it easier to convert a biological event into an electronic signal, which is a key challenge in biosensor design [34].

While these sensors show great promise, challenges like maintaining stability and accuracy in complex biological samples remain. Future work may focus on improving polymer materials and combining them with new technologies for better performance. In summary, conducting polymers provide a versatile and effective platform for both gas and microbial biosensing. Their unique traits make them central to developing smart sensors capable of detecting a wide range of chemical and biological targets, useful in many real-world applications such as environmental monitoring, health, and food safety.

This work proposes a novel SOI-TFET-based sensing platform that strategically addresses key design limitations while leveraging the multifunctionality of conducting polymers for both gas and microbial biosensing. The following sections detail the sensor’s design, fabrication, simulation methodology, and performance evaluation, highlighting its potential for real-time, low-power, and highly selective detection in diverse application spaces.

## 2. Proposed Gas Sensor Structure

**Figure 1:**
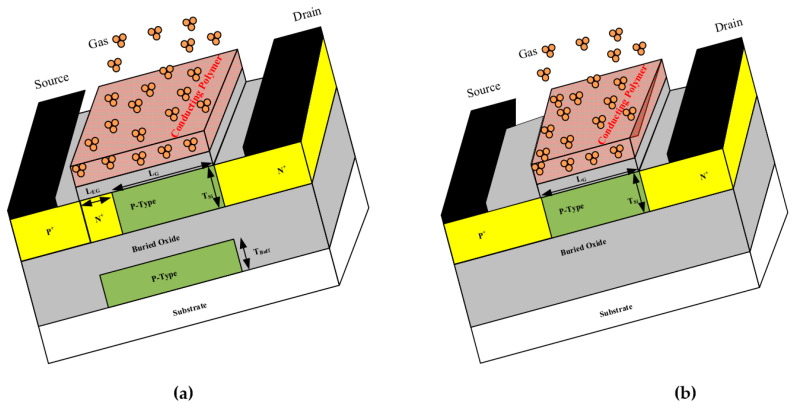
A schematic of the gas sensors under study implemented in this work. See (**a**) for the proposed gas sensor and (**b**) for the conventional gas sensor. The conventional sensor lacks the buffer region, extra doping inside the source, and extended gate as compared to the proposed sensor.

The core of the proposed gas sensor is a single-gate SOI-TFET device. To increase sensitivity and selectivity, three important modifications have been implemented: gate electrode engineering, doping engineering, and self-heating management, resulting in the design of the proposed gas sensor, as demonstrated in Figure 1a. The sensing performance of the proposed gas sensor has been compared by that of a conventional gas sensor illustrated in Figure 1b for more clarification. The first modification in the proposed sensor relative to the conventional sensor involves inserting an n-type extra doping region with a width of 5 nm inside the source region. By shifting the peak electric field from the source/channel interface to the source/extra-doping region interface, the effective tunneling width is reduced, which enhances the band-to-band tunneling rate and leads to a higher drain current. The second change is extending the gate length into the source region to completely overlap with the extra doping region, thereby applying strong electrostatic coupling and reducing the subthreshold swing. This feature is particularly valuable for gas and microbial biosensing, where fast and low power switching responses are critical. As the third change, a p-type buffer region is introduced into part of the buried oxide to act as a heat sink, thereby increasing thermal conductivity and reducing the lattice temperature.

To enhance the selectivity of the gas sensor, the proposed structure utilizes conducting polymers as sensing elements. The sensor’s performance has been evaluated using various polymer groups, including PPP-TOS/AcCN, PP-TOS/AcCN, PP-FE(CN)63-/H_2_O, PPP-TCNQ-TOS/AcCN, and PPP-CIO4/AcCN. These deposited organic polymers are based on polypyrrole (PP) and poly(p-phenylene) (PPP) films, which serve as the core materials in forming organic hydrocarbon chains. When these polymers act as sensing elements, analyte gases such as methanol (CH_3_OH), chloroform (CHCl_3_), isopropanol (i-C_3_H_7_OH), and hexane (n-C_6_H_14_) are absorbed into the conducting polymers. The formation of charge transfer complexes between the gas analytes and organic polymers leads to a modification of the gate workfunction. This modulation directly impacts the channel conductance, forming an electrical basis for detection. The same mechanism can be extended to microbial biosensing, where metabolic byproducts interact with the polymer gate, altering its electronic properties. This multi-functionality makes the proposed structure a dual-mode sensor suitable for both chemical and biological targets. Essential parameters for simulation of the proposed gas sensor are mentioned in Table 1.

**Figure 2:**
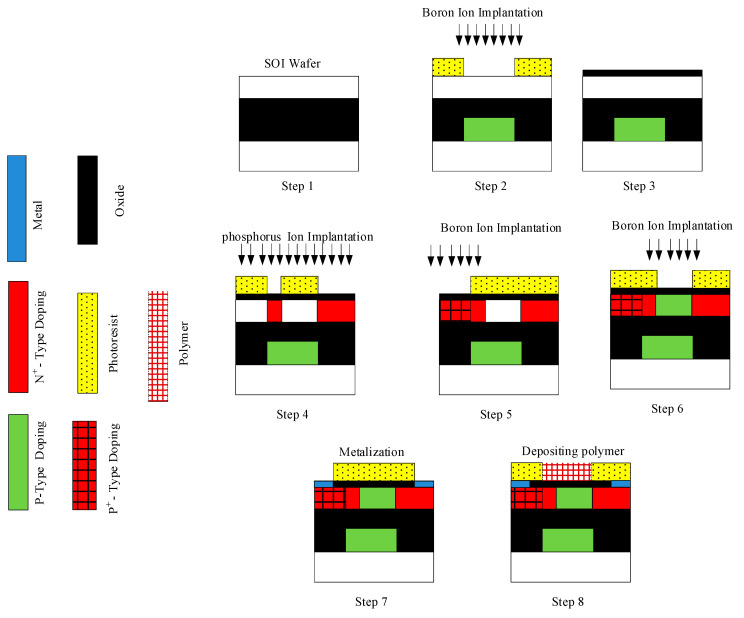
The fabrication steps of the proposed gas sensor.

In the rest of the work, the fabrication steps of the proposed gas sensor have been proposed by demonstrating them in Figure 2. Eight proposed steps are introduced as the subsequent statements [18,20,35,36]:

Step 1: Begin with a silicon-on-insulator (SOI) wafer consisting of a 10 nm lightly p-type silicon device layer, a 25 nm buried oxide (BOX), and a silicon substrate as indicated in step 1. Clean the wafer using the RCA process to ensure a contaminant-free surface, which is critical for high-quality device fabrication.

Step 2: Spin-coat photoresist and use photolithography to open a window in the BOX where the buried p-type buffer will be formed. Employ reactive ion etching (RIE) to etch the BOX to a depth of 12.5 nm only in the patterned regions. Perform boron ion implantation (dose: 1 × 10^13^ cm^−2^, energy: 30–50 keV) through these windows, and then strip the resist. Activate the dopants and repair damage with rapid thermal annealing at 950 °C for 10 s, resulting in the green p-type region within the BOX.

Step 3: Grow or deposit a 1 nm SiO**_2_** gate oxide layer over the entire wafer using rapid thermal oxidation or atomic layer deposition (ALD), providing a high-quality gate dielectric essential for controlling the channel.

Step 4: Spin-coat photoresist and use photolithography to open windows for the N^+^ regions (drain and extended gate). Implant phosphorus or arsenic ions (dose: 1 × 10^15^ cm^−2^, energy: 2–5 keV) to achieve N^+^ doping (1 × 10^20^ cm^−3^), and then remove the resist. Activate the dopants with RTA at 1000 °C for 5 s, forming the yellow N^+^ regions.

Step 5: Spin-coat photoresist and use photolithography to open a window only over the source region. Implant boron ions (dose: 1 × 10^15^ cm^−2^, energy: 2–5 keV) to achieve P^+^ doping (1 × 10^20^ cm^−3^). Remove the resist and anneal at 1000 °C for 5 s to activate the dopants, forming the P^+^ source region.

Step 6: Spin-coat photoresist and use photolithography to open a window over the channel region. Implant boron ions (dose: 1 × 10^12^ cm^−2^, ~2 keV) to achieve a lightly p-type channel (1 × 10^17^ cm^−3^). Strip the resist and anneal at 800 °C for 10 s to activate the dopants, ensuring a sharp channel profile.

Step 7: Spin-coat photoresist and use photolithography to define openings for source and drain contacts. Deposit a blanket layer of Ni or Ti (10–30 nm) by sputtering. Use lift-off to leave metal only in the patterned contact regions. For Ni, anneal at 400 °C for 30 s to form low-resistance NiSi contacts.

Step 8: Clean the gate oxide surface with O**_2_** plasma or piranha solution to remove organic residues. Apply a silane self-assembled monolayer (SAM) to improve adhesion of the polymer gate. Spin-coat photoresist and use photolithography to define the gate area. Spin-coat a PPP-TOS/acetonitrile solution (10 mg/mL) at 3000 rpm for 40 s, bake at 70 °C for 2 min and 110 °C for 10 min in N**_2_**, and then lift off unwanted polymer. At the end, deposit a passivation layer (SiN or SiO**_2_**, ~50 nm) by PECVD, and then use RIE to open the sensing window. Strip the resist after etching, leaving the polymer gate exposed for gas sensing.

## 3. Numerically Computational Methodology

This section presents the computational framework employed for the numerical simulation of the proposed gas sensor. The energy balance equation is solved to accurately describe carrier transport phenomena, essential for modeling tunneling-dominant SOI-TFET behavior under chemical and microbial biosensing scenarios. Given that the sensor operates at the nanoscale, the energy balance equation is solved to accurately model carrier transport within the simulation domain. The simulation incorporates two charge transport mechanisms, thermionic emission and band-to-band tunneling (BTBT), which are integrated to provide precise predictions of the device’s electrical behavior. Both local and non-local tunneling phenomena are considered throughout the simulation domain.

To capture the influence of local potential variations on the energy profile, the Poisson equation is solved self-consistently alongside the transport equations. Carrier dynamics for both electrons and holes are evaluated at each grid point within the device structure. The fundamental governing equations used in this simulation framework are presented in the notation below [37].

(1) $$\vec{J_{n}}=\;qD_{n}\nabla (n)-q\mu_{n}n\nabla \psi +qn{D_{n}}^{T}\nabla T_{n}$$

(2) $$\vec{J_{p}}=-qD_{p}\nabla p-qp\mu_{p}\nabla \psi +\mu_{p}p(kT_{L}\nabla (Lnn_{ie}))$$

(3) $$div\left(\vec{S_{n}}\right)=(1/q)\vec{J_{n}}.\vec{F}-M_{n}-(3/2)k\partial (\lambda_{n}nT_{n})/\partial t$$

(4) $$div\left(\vec{S_{n}}\right)=(1/q)\vec{J_{n}}.\vec{F}-M_{n}-(3/2)k\partial (\lambda_{n}nT_{n})/\partial t$$

(5) $$\vec{S_{n}}=-k_{n}\vec{\nabla }T_{n}-(k\alpha_{n}/q)\vec{J_{n}}T_{n}$$

(6) $$\partial n/\partial t=(1/q)div(\vec{J_{n}})+G_{n}-R_{n}$$

(7) $$\partial p/\partial t=-(1/q)div(\vec{J_{p}})+G_{p}-R_{p}$$

(8) $$div\left(\varepsilon \nabla \psi \right)=-\left(N_{D}+P-N_{A}-n\right)$$

It is important to note that the variables $\mu_{n}$ and $D_{n}$ denote the effective mobility and diffusion constant of electrons, respectively. The subscript p refers to the corresponding parameters for holes, while n indicates those for electrons. Here, q represents the elementary charge, K is the Boltzmann constant, and T_L_ denotes the local lattice temperature. The terms G_n,p_ and R_n,p_ correspond to the generation and recombination rates of electrons and holes. The current densities for electrons and holes are expressed as J_n_ and J_p_. The electrostatic potential, doping concentration, acceptor concentration, electron density, and hole density across the simulation domain are represented by $\psi ,N_{D},N_{A},n,p$, respectively. To account for self-heating effects in the SOI-TFET, the heat equation is formulated according to the following relation:

(9) $$C\partial T_{L}/\partial t\;=\;\nabla \left(k\nabla T_{L}\right)+H$$

To consider the local and non-local tunneling rate, the equations below are given in the simulation:

(10) $$G_{Klassen}=\;\alpha E^{\beta }\exp\left(-\frac{\lambda }{E}\right)$$

(11) $$J\left(E\right)=\;\frac{q}{\pi \hbar }\iint T\left(E\right)\left[f_{l}\left(E+E_{T}\right)-f_{r}\left(E+E_{T}\right)\frac{\sqrt{m_{e}m_{h}}}{2\pi \hbar^{2}}\right]dEdE_{T}$$

(12) $$f_{l}={\left(1+\exp(E+E_{T}-E_{Fl})/KT\right)}^{-1}$$

(13) $$f_{r}={\left(1+\exp(E+E_{T}-E_{Fr})/KT\right)}^{-1}$$

The term G_Klassen_ represents the local tunneling contribution incorporated into Equation (6), while J(E) signifies the non-local tunneling associated with the initiation and termination tunneling points within the grid points of the source region. Comprehensive details regarding these terms are provided in [33].

For numerical solution of the coupled system, appropriate boundary conditions must be rigorously applied at structural interfaces. Specifically, Neumann and Dirichlet boundary conditions are imposed throughout the simulation domain. The governing equations are discretized using the finite box method, enabling efficient extraction of device characteristics [38]. To enhance numerical stability and convergence, the Newton–Raphson iterative algorithm is employed, utilizing an initial guess for the potential profile. Simulations are conducted using the ATLAS device simulator within the SILVACO TCAD suite to iteratively solve the coupled equations (Equations (1)–(13)) [38].

To ensure physical accuracy, the simulator integrates several advanced models, including transverse electric field-dependent mobility, Shockley–Read–Hall (SRH) recombination, and bandgap narrowing to account for high doping effects, alongside Fermi–Dirac statistics.

Additionally, quantum confinement effects are rigorously incorporated in both device structures to enhance the fidelity of the simulation results.

**Figure 3:**
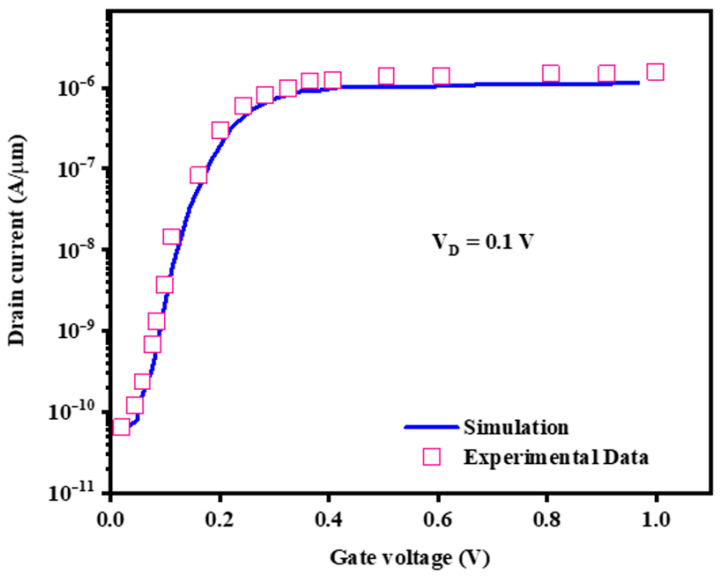
The calibration of utilized simulator by experimental TFET-based data [17].

Since the sensor is based on the TFET device, we have calibrated the software with experimental data relying on the TFET structure governed by non-local band-to-band tunneling [17]. Figure 3 demonstrates the transfer characteristics for both experimental data and numerically obtained data by software. Showing in the figure, there is an excellent match between the experimental and numerical data.

The simulation of a tunnel field-effect transistor (TFET) with metal and conducting polymer gates as a gas sensor was calibrated against experimental results using polypyrrole-based gates [39]. The simulated device achieved a subthreshold swing of 11.2 mV/dec, closely matching the experimental 13 mV/dec at a gas concentration of 4.6 × 10^−3^ mol dm^−3^. This validates the simulation parameters and confirms the transistor’s feasibility for gas sensing applications. By applying this robust simulation framework, we capture not only the quantum transport behavior necessary for trace gas detection but also extend the model’s relevance to complex microbial biosensing environments.

## 4. Synthesis Details of Conducting Polymers

Conductive polymers such as poly(p-phenylene) (PPP) and polypyrrole (PP) have become pivotal in high-performance gas sensor technologies due to their easily tunable electrical properties, ease of synthesis, and strong interactions with gas molecules [1]. These polymers are typically synthesized via chemical or electrochemical polymerization, where the choice of dopant and solvent plays a critical role in determining the final conductivity and sensing performance. These doping strategies significantly influence polymer morphology and electrical behavior, enabling rapid, selective responses to gas-phase analytes and bio-targets alike. The same structural and electronic modifications that enhance gas interaction also support microbial biosensing, where microbial metabolites can induce comparable shifts in the polymer’s electronic profile.

**Figure 4:**
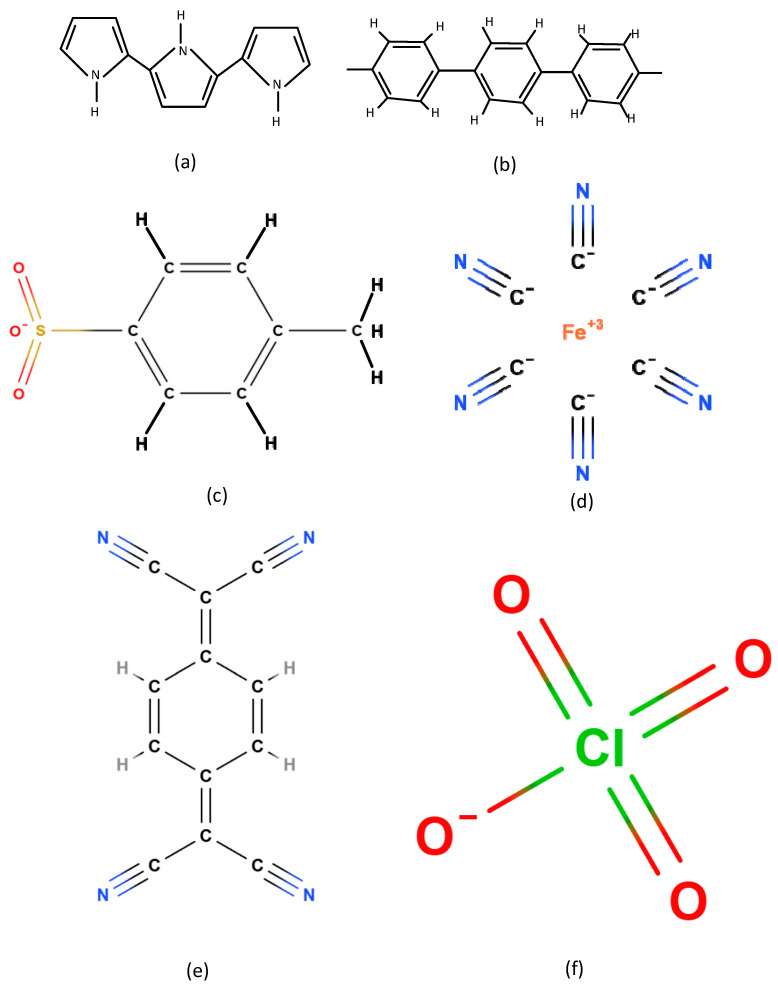
A visualization of conducting polymers implemented as sensing element in the proposed sensor in the cases of (**a**) polypyrrole (PPy) and (**b**) p-polyphenylene (PPP). (**c**) TOS in PPP-TOS/AcCN polymers and (**d**) Fe(CN)_6_^3−^ in PP-FE(CN)_6_^3−^/H_2_O as well as (**e**) TCNQ in PPP-TCNQ-TOS/AcCN and (**f**) ClO_4_^−^ in PPP-ClO_4_/AcCN are illustrated.

**Figure 5:**
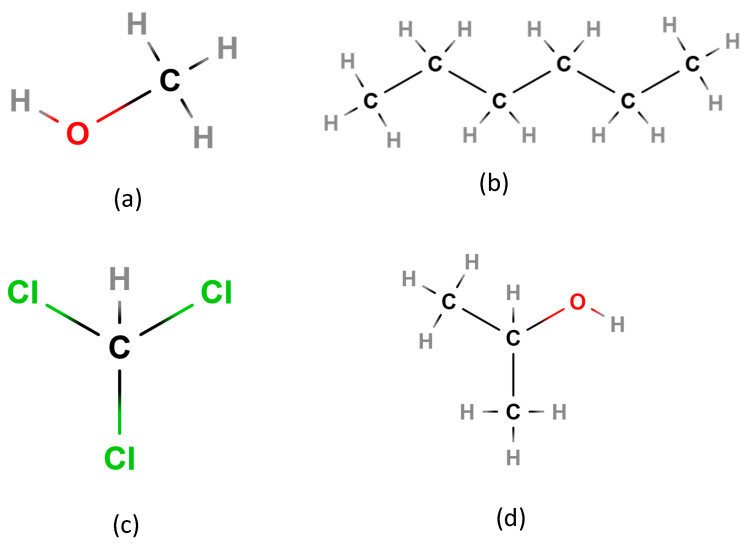
Molecular structure of exposed gases into the conducting polymers such as (**a**) methanol, (**b**) hexane, (**c**) chloroform, and (**d**) isopropanol.

Figure 4 and Figure 5 illustrate the polymer structures and the analyte molecules, respectively, emphasizing how their interaction at the molecular level translates to electrical modulation within the sensor. This dual-sensing compatibility highlights the polymers’ adaptability and strengthens the sensor’s versatility for both environmental and biological monitoring. Polypyrrole (PP) consists of repeating pyrrole units ([-C_4_H_2_NH-]n), each being a five-membered ring with four carbons and one nitrogen, joined at their α-positions, creating a linear, π-conjugated backbone that enables high conductivity [40] as illustrated in Figure 4a. In contrast, p-polyphenylene (PPP) features repeating benzene rings linked at the para (1, 4) positions, yielding a stiff, planar chain with repeat unit [-C_6_H_4_-]n, as visualized in Figure 4b [41]. In PPP-TOS/AcCN, PPP is formed by electrochemically polymerizing phenyl monomers in acetonitrile (AcCN) with tosylate (TOS^−^) as a dopant, which boosts conductivity and stability for sensitive gas sensing, as shown in Figure 4c [42]. PP-TOS/AcCN is similarly synthesized, resulting in PP films with rapid, room-temperature sensor response [41]. For PP-FE(CN)_6_^3−^/H_2_O, ferricyanide (Fe(CN)_6_^3−^) doping adds redox properties and selectivity, as seen in Figure 4d. PPP-TCNQ-TOS/AcCN uses dual doping (TCNQ and TOS^−^) to tailor PPP’s electronic structure and sensing specificity, as visualized in Figure 4e [41]. PPP-ClO_4_/AcCN employs perchlorate (ClO_4_^−^) to further tune conductivity, as visualized in Figure 4f. These doping and structural strategies enable control over morphology and electrical properties, resulting in sensors with high sensitivity, selectivity, and fast room-temperature response.

PP and PPP films are prepared using a potentiostat [43]. For PP films, a series of voltage pulses (0.9 V for 0.1 s, then off for 1 s) were applied repeatedly until 100 mC/cm^2^ of charge had passed. The resulting PP films are about 500 nm thick, measured using a profilometer. For PPP films, deposition followed a procedure from Ashley et al., where electrodes were cycled between 1.0 and 1.8 V at a rate of 100 mV/s [44]. Using perchlorate as the supporting electrolyte, the full 100 mC/cm^2^ charge passed and produced films around 40 nm thick. When TOS-anions were used, only about 45 mC/cm^2^ of charge flowed before the film’s resistance stopped the process; these films are too thin to measure. The PPP films could not be grown successfully on ITO substrates. Because organic solvents can cause high resistance between electrodes, IR compensation is used during all depositions. Interestingly, PP films are about 10 times thicker than PPP films for the same amount of charge. This may be because a lot of formed PPP dissolves in the solvent as soluble oligomers and does not deposit as a solid film. After deposition, all films are rinsed with plain solvent and air-dried for at least 24 h before use.

To generate organic vapors, nitrogen gas is bubbled through the liquid compound at room temperature. The vapor flow is diluted with more nitrogen and its concentration is measured by gas chromatography. The detected vapor concentrations are methanol: 4.6 × 10^−3^ mol/dm^3^ (112,470 ppm), isopropanol: 0.8 × 10^−3^ mol/dm^3^ (9780 ppm), n-hexane: 1.6 × 10^−3^ mol/dm^3^ (39,120 ppm), dichloromethane: 8.5 × 10^−3^ mol/dm^3^ (207,825 ppm), and chloroform: 1.2 × 10^−3^ mol/dm^3^ (29,340 ppm). Samples are alternately exposed to either pure nitrogen or the organic vapor stream. UV/visible absorption measurements of the films are recorded using a spectrometer. Photoelectrochemical tests are performed under controlled voltage using a special cell filled with background electrolyte solution, similar to conditions used in film deposition.

## 5. Different Gas Analytes

This work evaluates the performance of the proposed sensor against four industrially significant gas analytes: methanol (CH_3_OH), chloroform (CHCl_3_), isopropanol (i-C_3_H_7_OH), and hexane (n-C_6_H_14_). These compounds are commonly encountered in environmental, laboratory, and manufacturing settings, and each poses distinct health and safety risks upon inhalation.

Methanol gas (CH_3_OH/MeOH) is a colorless, highly flammable vapor with a faintly sweet odor; inhalation can lead to severe toxicity, including nervous system damage and even death [45,46]. Chloroform vapor (CHCl_3_) is dense, non-flammable, and can induce dizziness or anesthesia at high concentrations. Isopropanol (i-Propanol) vapor is flammable, has a strong odor, and may cause narcotic effects when inhaled in large quantities. Hexane gas (C_6_H_14_) is volatile, highly flammable, non-polar, and neurotoxic with prolonged exposure. While all four analytes have practical industrial applications, their potential hazards demand detection at very low concentrations. The proposed sensor, utilizing polymer-coated gates, detects these vapors through modulation of the gate workfunction via analyte–polymer charge-transfer interactions. This same sensing principle is translatable to microbial biosensing, where volatile organic compounds (VOCs) and metabolic byproducts emitted by microbes similarly alter the electronic properties of conducting polymers. Thus, characterizing sensor response to volatile chemicals also supports its utility in microbial diagnostics. The molecular structures of these analytes are shown in Figure 3, providing a visual basis for understanding their interaction potential with the polymer matrix.

## 6. Mechanism of Gas-Driven Electron Transfer in Polymers

A donor molecule, such as gas G, transfers to or accepts from the polymer matrix a fraction of its electronic charge upon entry, according to the following relation [47]:

(14) $$G\;=\;G^{\delta }+\delta e$$

This charge-transfer equilibrium, as given in Equation (15), is defined by the equilibrium constant K_G_, the partial pressure of gas P_G_, and the solubility α, with the dissolved gas concentration given as P_G_.

(15) $$K_{G}={\left[e\right]}^{2\delta }/\alpha P_{G}$$

The ease of ion formation by the molecule depends on the average of its electron affinity E_a_ and ionization potential I_p_, known as Mulliken electronegativity, with χ defined in the equation below:

(16) $$\chi =\;0.5(E_{a}-I_{p})$$

The semiconductor’s ability to transfer electrons is linked to its Fermi level E_F_, and fractional charge transfer δ depends on the difference between χ and E_F_ as indicated in the following:

(17) $$\delta =\;\zeta (E_{F}-\chi )$$

Hence, fractional charge transfer results in a change in the gate conducting polymer workfunction, directly impacting the band energy profile. In this study, the polymers were exposed to vapor streams of methanol (CH3OH/MeOH, 4.6 mM or 112,470 ppm), isopropanol (i-C3H7OH, 0.8 mM or 9780 ppm), n-hexane (n-C6H14, 1.6 mM or 39,120 ppm), and chloroform (CHCl3, 1.2 mM or 29,340 ppm), delivered in a nitrogen carrier [48]. The vapor concentrations were measured and confirmed using gas chromatography. Vapor concentrations were precisely quantified using gas chromatography. Upon exposure to each vapor, the water flux (WF) stabilized, reaching equilibrium within 60 s, indicating rapid polymer response to the different chemical environments, a critical feature for real-time monitoring applications in both environmental and biosensing domains.

This electron-transfer mechanism is equally applicable to microbial biosensing, where microbial byproducts, including aldehydes, ketones, and acids, possess electron-rich or electron-deficient structures capable of inducing similar gate workfunction shifts. As such, the same physics governs both chemical gas detection and biological metabolite sensing.

Workfunction modulation occurs when the charge is transferred between the polymer and gas molecules. The variation magnitude of workfunction ($\Delta W_{F}$) depends on both the polymer and gas molecules. Table 2 has listed the value of workfunction shift for PPP-TOS/AcCN conducting polymer by reference of experimentally extracted Au metal [43,48]. It is pointed out that that workfunction modulation changes by different conducting polymers. The extra information utilized for other polymers has been provided in [43,48].

## 7. Discussion on Simulated Results

This section is attributed to theoretically modeling the gas sensors under study. All the extracted results are based on the theoretical analysis. Calibrating the simulation software, the magnitude of workfunction shift utilized in the paper is based on the experimentally provided information by [43,48].

**Figure 6:**
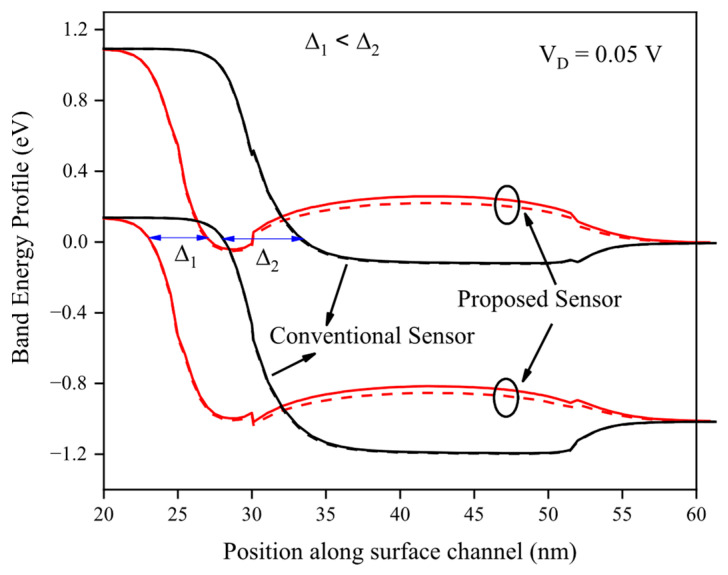
The energy profile along the surface channel for the proposed and conventional gas sensors. The drain voltage has been fixed at the bias of 0.05 V.

The band energy profiles along the surface channel at a drain voltage of 0.05 V reveal that the proposed gas sensor, utilizing PPP-TOS/AcCN as the sensing polymer, experiences a significant reduction in both the energy barrier and the tunneling width (Δ_1_) upon exposure to methanol (MeOH) gas molecules, in contrast to the conventional gas sensor, as illustrated in Figure 6. These reductions enhance electrostatic modulation within the channel, effectively suppressing the subthreshold swing and facilitating more efficient carrier transport. Such modulation results in significantly heightened sensitivity in the proposed sensor, confirming its superior ability to transform gas-induced chemical interactions into pronounced electrical responses at low power.

**Figure 7:**
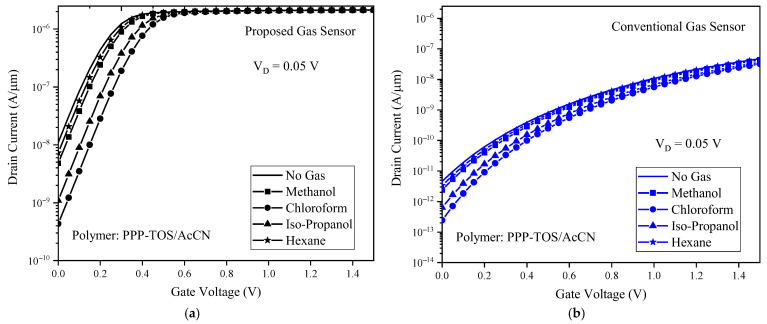
Drain current versus the gate voltage for (**a**) proposed gas sensor (**b**) conventional gas sensor. The PPP-TOS/AcCN polymer is exposed to different gases.

Comparative analysis of the drain current versus gate voltage at V_ds_ = 0.05 V for the proposed (Figure 7a) and conventional (Figure 7b) gas sensors demonstrates that the proposed sensor delivers markedly higher ON-state drain current alongside a reduced subthreshold swing, amplifying its detection efficiency for the detection of methanol, chloroform, isopropanol, and hexane. These performance advantages arise from reduced energy barriers and enhanced gate control, both characteristic of the proposed SOI-TFET design. Notably, the low subthreshold swing reduces noise, which is critical in TFETs that inherently operate at low current levels, thereby enhancing reliability and minimizing signal ambiguity. As a result, high sensitivity is achieved alongside ultra-low power consumption, as even modest biasing is sufficient to produce pronounced current variations upon gas exposure, a highly desirable feature for practical and energy-efficient gas sensing platforms.

Time response is a crucial factor in both biosensors and gas sensors, as it determines how quickly the sensor detects changes and stabilizes, enabling real-time monitoring [49]. Ensuring high sensitivity while maintaining a fast time response is vital for accurate and timely detection. According to Equation (18), there is a direct dependence of time response (t_r_) on subthreshold swing, where a lower subthreshold swing leads to faster sensor dynamics [50].

(18) $$t_{r}=K\frac{{SS}_{avg}}{\rho_{0}}\;$$

where parameter K is a process related constant, and SS_avg_ and $\rho_{0}$ are the average subthreshold swing and gas molecule concentration, respectively. In the same sensitivity, the proposed gas sensor achieves a reduced subthreshold swing compared to conventional sensors, resulting in a significantly faster time response without sacrificing sensitivity.

**Figure 8:**
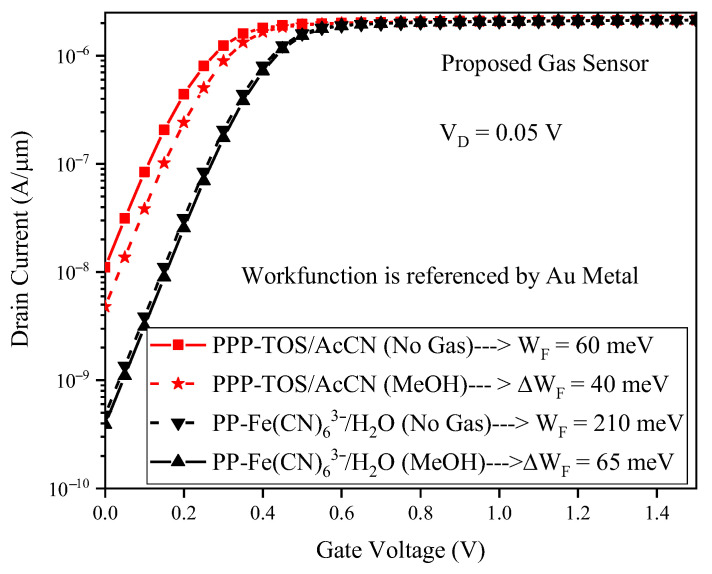
Drain current versus the gate voltage for the proposed gas sensor. MeOH gas is exposed to the polymers including PPP-TOS/AcCN and PP-Fe(CN)63-/H_2_O.

Figure 8 compares the proposed sensor’s response to MeOH detection using two conducting polymers: PPP-TOS/AcCN and PP-Fe(CN)63-/H_2_O. PPP-TOS/AcCN produces a substantially greater change in drain current upon gas exposure, attributable to its higher charge transfer efficiency with MeOH analytes. This signifies enhanced interaction at the polymer/gas interface, directly amplifying sensitivity. Therefore, careful selection of the sensing polymer, as investigated comprehensively throughout this study, is instrumental in customizing gas sensor performance, distinguishing this research from prior works by providing practical polymer selection guidelines for diverse analytes.

To measure the sensing power in detecting the gas analytes, two metrics have been used. The first one is the threshold voltage sensitivity (S_VTH_) pointing to the difference between the threshold voltages before and after the injection of gas molecules as defined in the following relation:

(19) $$S_{VTH}=\;\left|V_{TH,before}-V_{TH,after}\;\right|$$

The other definition of sensitivity highlighting on the OFF current has been selected in order to reach a tradeoff between the sensitivity and selectivity as given in the subsequent form:

(20) $$S_{OFF}=\;\left|\frac{I_{OFF,before}}{I_{OFF,after}}\right|$$

It is worth noting that OFF current is evaluated in gate voltage of 0 V.

**Figure 9:**
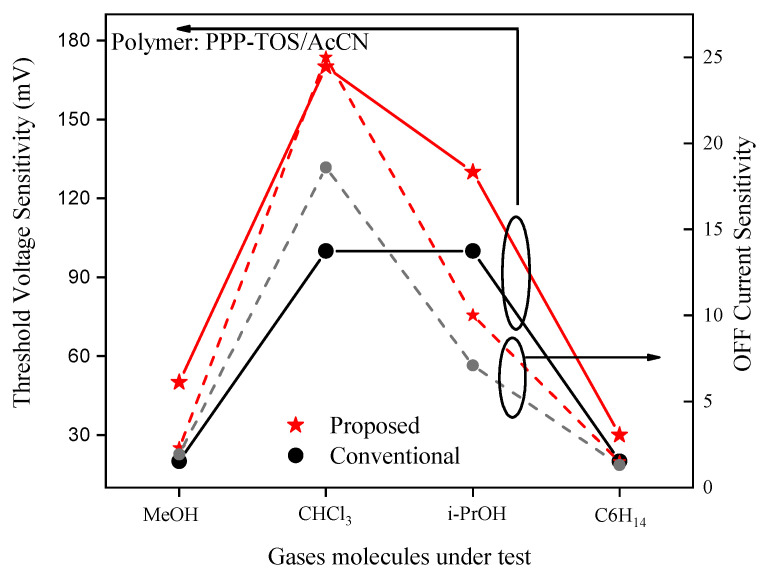
Threshold voltage and OFF current sensitivities for both the proposed and conventional gas sensors.

The analysis of sensitivity metrics—threshold voltage and OFF current sensitivities—across MeOH, CHCl_3_, i-PrOH, and C_6_H_14_ at V_ds_ = 0.05 V in both conventional and proposed sensors underscores complementary performance, as illustrated in Figure 9. Both metrics follow a similar trend, peaking at CHCl_3_ and declining thereafter, though threshold voltage sensitivity consistently surpasses OFF current sensitivity in magnitude for both devices. Selectivity, as indicated by the derivative of sensitivity with respect to gas type, is higher when OFF current sensitivity is used as the metric, particularly in cases where analytes more strongly affect the OFF current. However, the conventional sensor shows reduced selectivity between chloroform (CHCl_3_) and isopropanol (i-PrOH), as the threshold voltage sensitivity for these gases converges. As a meaningful result, it is observed from the figure that the proposed gas sensor exhibits greater threshold voltage and OFF current sensitivities than the conventional sensor, indicating its promise as a highly sensitive gas detection platform. This dual-metric approach provides a more nuanced understanding of gas-sensing mechanisms, enhancing the applicability of the sensor to varied environments.

**Figure 10:**
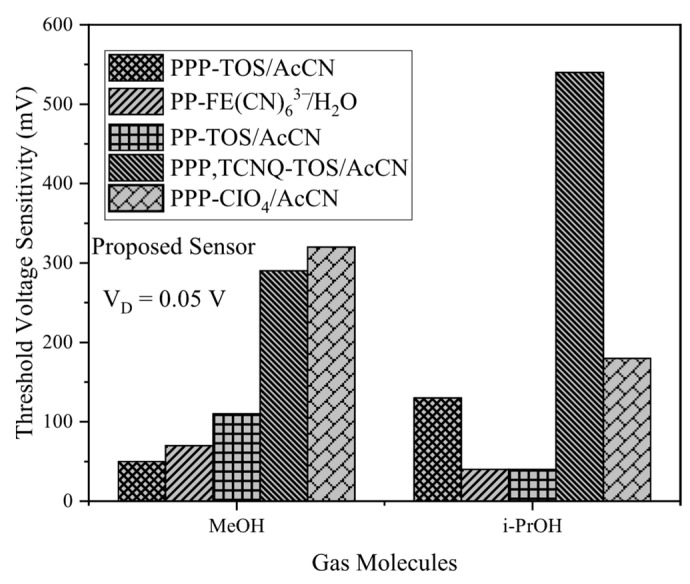
Threshold voltage sensitivity of the proposed gas sensor including 5 different conducting polymers. MeOH and i-PrOH gas molecules are exposed to these polymers.

Threshold voltage sensitivity measurements for the proposed gas sensor with MeOH and i-PrOH using polymers, PPP-TOS/AcCN, PP-Fe(CN)_6_^3−^/H_2_O, PP-TOS/AcCN, and PPP-CIO_4_/AcCN, as demonstrated in Figure 10, reveal high sensitivity and selectivity across the board. Among these, PPP-TCNQ-TOS/AcCN exhibits outstanding selectivity for i-PrOH, making it the optimal choice for this target, while PP-TOS/AcCN and PPP-CIO_4_/AcCN prove most effective for MeOH detection. These outcomes facilitate targeted polymer selection for maximizing sensitivity in specific gas detection applications.

**Figure 11:**
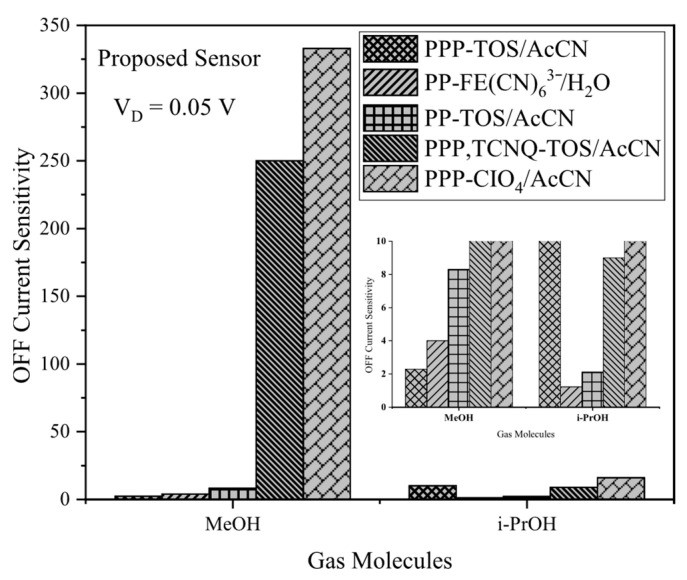
OFF current sensitivity of the proposed gas sensor including 5 different conducting polymers. MeOH and i-PrOH gas molecules are exposed to these polymers.

Analysis of OFF current sensitivity for MeOH and i-PrOH using the same polymer set visualized in Figure 11 shows that PPP-CIO_4_/AcCN and PPP-TCNQ-TOS/AcCN deliver strong, acceptable sensitivity for MeOH, but substantially lower values for i-PrOH, highlighting pronounced selectivity. The enlarged inset highlights the disparity, emphasizing that while threshold voltage sensitivity provides robust detection for both gases, OFF current sensitivity can be extremely specific, making it particularly effective for distinguishing methanol (MeOH) using these polymers. This leads to even greater selectivity than that achieved by threshold voltage sensitivity alone.

A critical comparison of Figure 10 and Figure 11 reveals that threshold voltage sensitivity enhances broad-spectrum gas detectability, ensuring general responsiveness, whereas OFF current sensitivity improves selectivity, particularly for PPP-TCNQ-TOS/AcCN and PPP-ClO_4_/AcCN polymers. Thus, a sensor’s application can be tuned depending on whether the priority is sensitive universal detection (threshold voltage) or high selectivity for specific analytes (OFF current).

**Figure 12:**
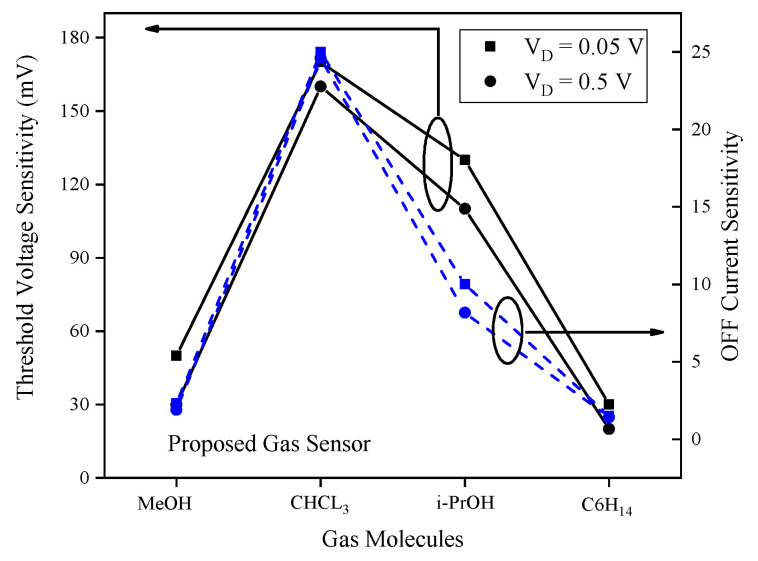
Illustration of sensitivity dependences of proposed gas sensor on the drain voltage.

For the proposed sensor using PPP-TOS/AcCN, evaluation of threshold voltage and OFF current sensitivities at drain voltages of 0.05 V (low-power mode) and 0.5 V (ON mode) across various gases reveals consistent trends in both operating regimes, as demonstrated in Figure 12. Threshold voltage sensitivity is more pronounced at lower bias, highlighting the advantages of energy-efficient operation in maximizing detection signal. In contrast, OFF current sensitivity remains largely unaffected by bias voltage, indicating its robustness as a sensing metric under varying operating conditions. Selectivity trends also remain stable across both voltages for each metric, enabling reliable sensor performance regardless of the applied bias.

**Figure 13:**
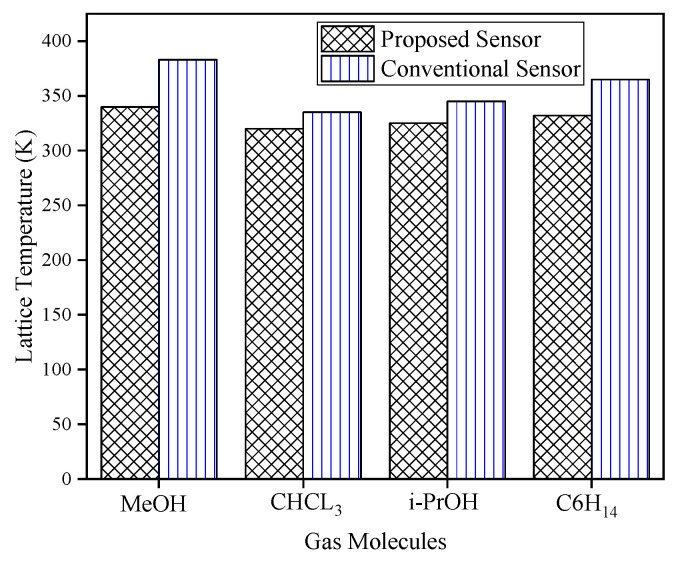
Lattice temperature versus different gas analytes for the proposed and conventional gas sensors.

Lattice temperature measurements for both conventional and proposed gas sensors demonstrate that the proposed design consistently achieves lower lattice temperatures across all tested gases, as shown in Figure 13. This reduction is attributed to structural innovation, specifically, the replacement of part of the buried oxide with a p-type buffer region, which enhances thermal conduction. This effective thermal management suppresses thermal instability, paving the way for more stable and reliable sensor operation under practical environmental fluctuations.

## 8. Design Considerations

Comprehensive investigations of threshold voltage and OFF current sensitivity as functions of multiple design parameters, including gate length, channel doping, source region doping, power supply, and buffer thickness, are rarely reported in the literature. Most existing works focus on the effects of individual parameters, thereby limiting opportunities for holistic optimization [51]. The use of the coefficient of variation (CV) as a reliability metric, quantifying the percentage deviation of sensitivity from its mean, represents a significant advancement, as previous studies typically lack rigorous and comprehensive reliability analyses in sensor design. By integrating multi-parameter exploration with statistical robustness evaluation, this work establishes a higher standard for sensitivity optimization and reliability prediction, offering substantial value and practical relevance beyond the standard, narrowly focused approaches commonly reported in the literature [1,52]. The conducting polymer of PPP-TOS/AcCN as a sensing element and the gas analyte of CHCL_3_ is assumed for the sensor evaluation in this section for the proposed gas sensor.

**Figure 14:**
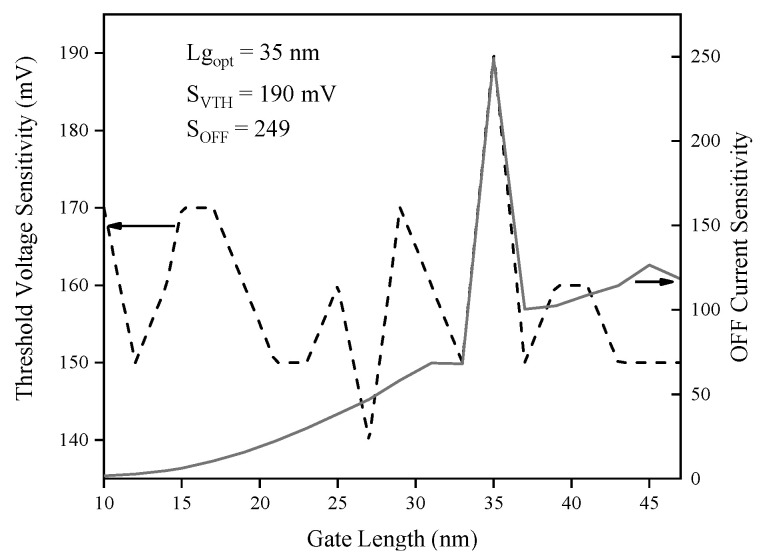
Threshold voltage and OFF current sensitivities versus gate length.

**Figure 15:**
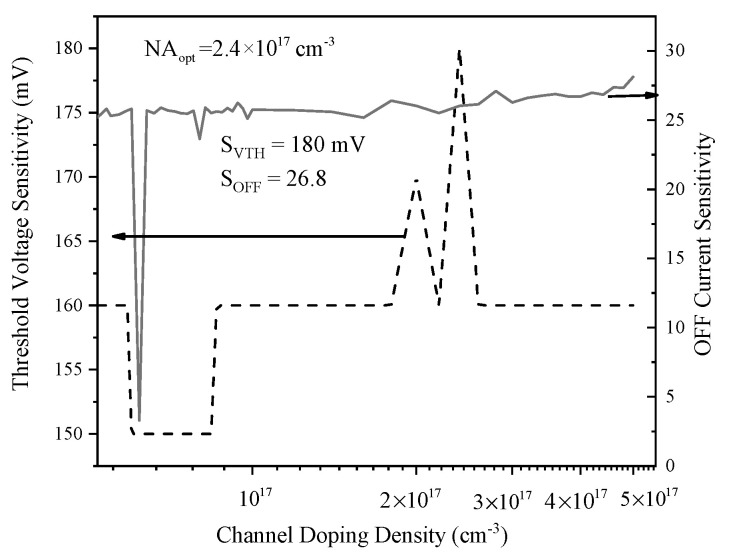
Threshold voltage and OFF current sensitivities versus channel doping density.

Figure 14 demonstrates that both threshold voltage sensitivity and OFF current sensitivity reach their maximum at a gate length (L_g_) of 35 nm, with S_OFF_ peaking at 249 and S_VTH_ at 190 mV, respectively, after fluctuating in the shorter gate length regime. As the gate length increases beyond this optimal point, both sensitivities decline, indicating reduced electrostatic control and diminished tunneling efficiency at larger dimensions. The pronounced peak at 35 nm signifies an optimal trade-off between enhanced sensitivity and device stability. Therefore, selecting Lg = 35 nm enables the sensor to leverage strong tunneling and electrostatic effects, making this value highly advantageous for maximizing gas detection performance and ensuring robust, reliable operation in practical SOI-TFET sensor applications. Figure 15 reveals an initial increase in threshold voltage sensitivity with rising channel doping density, attaining a maximum of approximately 180 mV at 2.4 × 10^17^ cm^−3^, then declining with further doping. In contrast, OFF current sensitivity remains nearly constant across the entire doping range, with a value of 26.8. This divergence implies that while threshold voltage sensitivity can be affected by variations in doping, OFF current sensitivity is inherently more stable with respect to channel doping fluctuations.

The optimal channel doping density of 2.4 × 10^17^ cm^−3^ is selected, as it maximizes threshold voltage sensitivity without sacrificing OFF current sensitivity, thereby securing high device responsiveness and minimizing the risk of excessive leakage or instability in operation that can be observed in realistic conditions.

**Figure 16:**
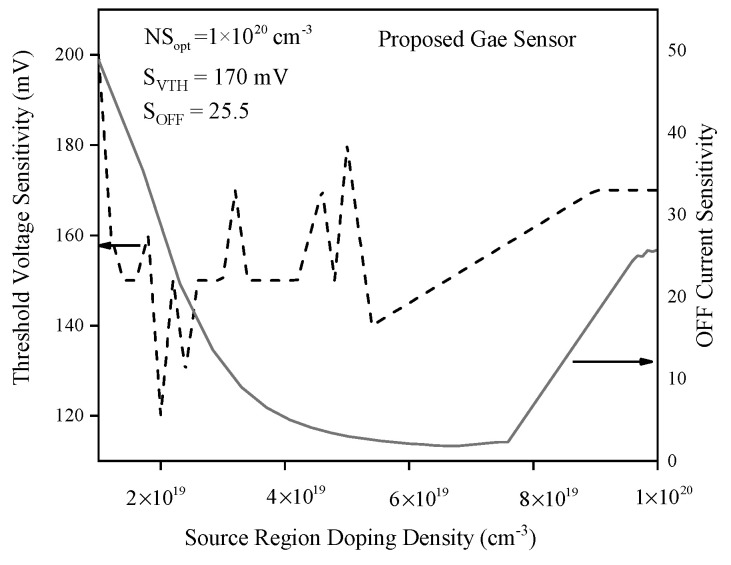
Threshold voltage and OFF current sensitivities versus source doping density.

Figure 16 presents a “bowl-shaped” OFF current sensitivity profile, with a minimum at a source doping density of 6 × 10^19^ cm^−3^, and maximum sensitivities observed at lower source doping. However, low doping cannot be considered optimal because the sensing current becomes overly controlled by the drain voltage, potentially compromising performance. Threshold voltage sensitivity shows irregular fluctuations at lower doping, followed by a monotonic increase at higher doping concentrations; the highest observed value is 170 mV at the maximum doping density of 1 × 10^20^ cm^−3^, where OFF current sensitivity is 25.5. Therefore, the maximum tested source region doping density is chosen as optimal, providing strong sensitivity and robust sensor operation by minimizing drain-voltage-induced variability and optimizing the tunneling junction for reliable gas detection.

**Figure 17:**
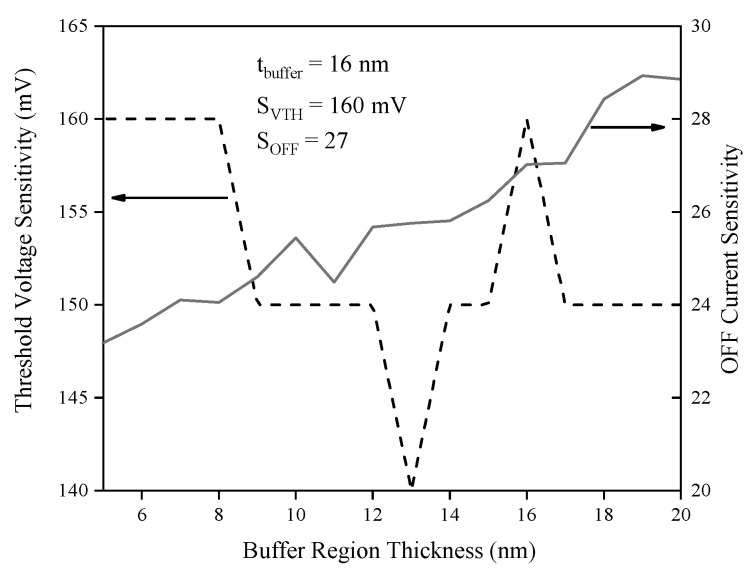
Threshold voltage and OFF current sensitivities versus buffer region thickness.

Figure 17 demonstrates that OFF current sensitivity increases continuously with buffer region thickness (t_Buff_), whereas threshold voltage sensitivity peaks sharply at 16 nm, where its value is 160 mV. Selecting a buffer thickness of 16 nm thus provides an optimal point, ensuring both high threshold voltage sensitivity and satisfactory OFF current sensitivity (27 at this thickness). This observation is due to favorable modulation of the electric field and minimized parasitic capacitance at this thickness, optimizing the tunneling condition necessary for enhanced sensor responsiveness and stable threshold operation.

**Figure 18:**
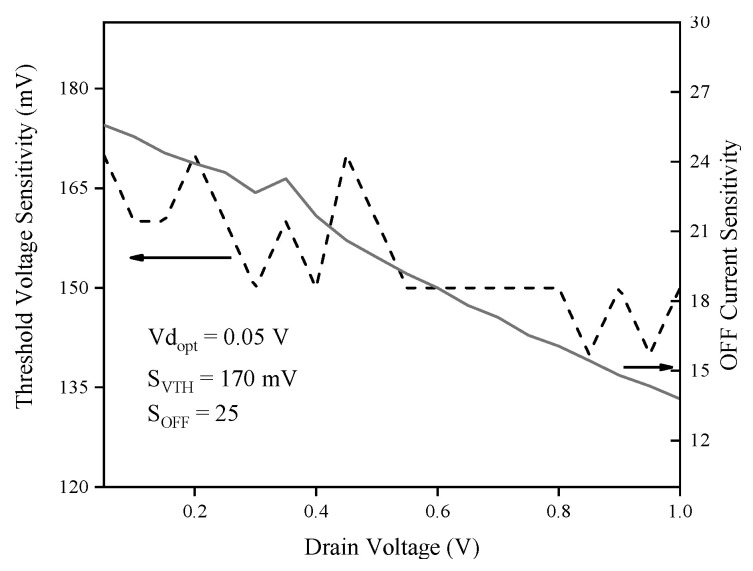
Threshold voltage and OFF current sensitivities versus drain voltage.

**Figure 19:**
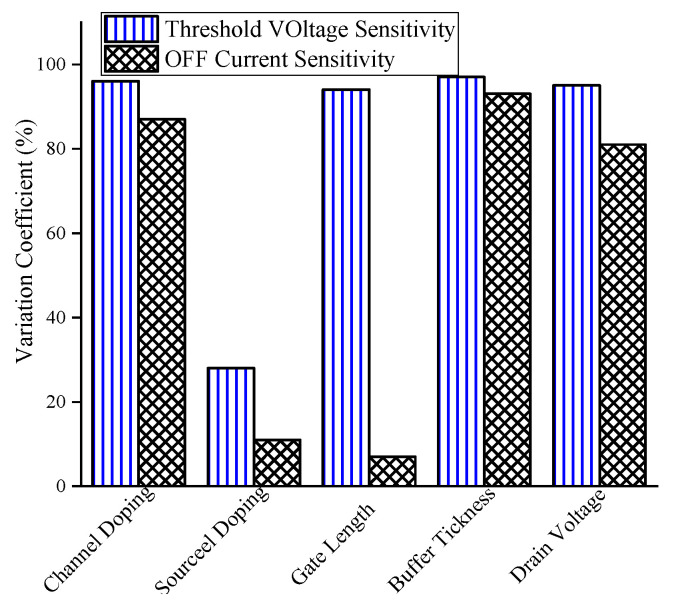
Illustration of variation coefficient versus different designing parameters based on both the sensitivities.

Figure 18 reveals that both sensitivities decline as drain voltage increases, with the highest values observed at a low drain voltage of 0.05 V, a condition crucial for achieving ultra-low-power sensor operation. At this voltage, threshold voltage sensitivity reaches 170 mV, while OFF current sensitivity is 25, indicating superior responsiveness in threshold-voltage-based detection. The low-voltage regime is therefore critical for maximizing sensitivity and minimizing power consumption, aligning with the energy-efficient design objectives of advanced gas sensor technologies. Figure 19 presents a statistical analysis using the coefficient of variation (CV) for threshold voltage sensitivity and OFF current sensitivity, plotted against channel doping, source doping, gate length, buffer thickness, and drain voltage. The CV is defined as follows in this work:

(21) $$CV=\left|\frac{\mu -\sigma }{\mu }\right|*100$$

where μ and σ are the average and standard divination of sensitivity upon the different ranges of designing parameters. According to the figure, both sensitivities exhibit comparable coefficients of variation (CVs) across most parameters, except for source doping and gate length. Notably, OFF current sensitivity shows a remarkably low CV with respect to gate length fluctuations, an important advantage for reproducibility given current nanoscale lithography limitations. In contrast, threshold voltage sensitivity displays slightly higher variability with gate length but generally offers greater absolute sensitivity. From a process-dependent perspective, OFF current sensitivity is preferable in applications where gate length uniformity is constrained, while threshold voltage sensitivity should be prioritized in scenarios requiring maximum detection precision.

Synthesizing information from Figure 12 through Figure 17 reveals a comprehensive guideline for sensor optimization. The analyses confirm that a gate length of 35 nm serves as the most favorable dimension for peak sensitivity, maximizing both threshold voltage and OFF current responses by optimizing the quantum tunneling conditions and electrostatic control. A moderate channel doping level of 2.4 × 10^17^ cm^−3^ ensures high threshold voltage sensitivity without destabilizing OFF current behavior. Maximum source doping (1 × 10^20^ cm^−3^) compensates drain voltage modulation, supporting robust threshold voltage and OFF current sensitivity. Buffer thickness optimization at 16 nm leverages electric field effects for peak performance. Critically, ultra-low drain voltage (0.05 V) substantially amplifies both sensitivities, fostering ultra-low power operation. The variation coefficient analysis underlines the importance of parameter selection based on process stability, favoring OFF current sensitivity where lithographic variability dominates and advocating threshold voltage sensitivity to achieve maximum sensing potential in controlled settings. Overall, this systematic multi-parameter optimization strategy harmonizes structural, process, and operational variables to deliver highly sensitive, highly selective, low-power, and robust SOI-TFET gas sensor platforms.

## 9. Conclusions

In this study, we have introduced a highly sensitive and versatile gas sensor platform based on a silicon-on-insulator tunneling field effect transistor (SOI-TFET) structure enhanced with engineered conducting polymers. Through a series of structural innovations including source-side doping, extended gate design, and thermal management via a p-type buffer, we achieved significant performance improvements over conventional gas sensors. The integration of tailored conducting polymers as gate materials has not only enabled selective detection of chemical analytes such as methanol, chloroform, isopropanol, and hexane but has also positioned the sensor as a promising candidate for microbial biosensing. The same electronic mechanisms that govern gas detection, namely, charge-transfer-induced modulation of the gate workfunction, are applicable to detect volatile microbial metabolites and other biologically relevant compounds. Dual sensitivity metrics, based on threshold voltage and OFF current analysis, were systematically explored and found to complement each other in balancing broad-spectrum responsiveness and target-specific selectivity. Furthermore, robustness under different biasing conditions and thermal stability ensures compatibility with a wide range of deployment scenarios, including portable and continuous monitoring systems. This work not only advances the field of TFET-based gas sensors but also lays a foundation for multifunctional sensing platforms capable of operating at ultra-low power while maintaining high resolution and reliability. Future efforts may extend this architecture to integrate real-time microbial detection in clinical diagnostics, food safety, and environmental biosurveillance applications.

## Figures and Tables

**Table 1:** Essential parameters for simulation of proposed gas sensor.

Parameters	This Work	Conventional
Gate length, L_G_	22 nm	22 nm
Extended gate length, L_EG_	5 nm	Not defined
Buffer region thickness, T_Buff_	12.5 nm	Not defined
Gate oxide thickness, T_Ox_	1 nm	1 nm
Si channel thickness, T_Si_	10 nm	10 nm
BOX thickness, t_Box_	25 nm	25 nm
Doping concentration of channel, N_A_	1 × 10^17^ cm^−3^	1 × 10^17^ cm^−3^
Doping concentration of source, N_S_	1 × 10^20^ cm^−3^	1 × 10^20^ cm^−3^
Extended region doping concentration, N^EG^	1 × 10^20^ cm^−3^	Not defined

**Table 2:** The value of workfunction modulation of PPP-TOS/AcCN polymer. The measurements are referenced to Au metal by workfunction of 5.1 eV.

Conducting Polymer: PPP-TOS/AcCN			
Gas Molecule	Initial workfunction, WF (meV)	Magnitude of Workfunction Modulation, (meV) $\Delta W_{F}$	Charge Transfer Type
MeOH	60	40	Acceptor
CHCl_3_	60	155	Acceptor
CH_2_Cl_2_	60	150	Acceptor
i-PrOH	60	110	Acceptor
C_6_H_14_	60	20	Acceptor

## Data Availability

The data is available upon request from the authors.
